# Benign Positional Paroxysmal Vertigo in Children

**DOI:** 10.3390/audiolres11010006

**Published:** 2021-02-01

**Authors:** Cristiano Balzanelli, Daniele Spataro, Luca Oscar Redaelli de Zinis

**Affiliations:** 1 Vertigo Center—San Bernardino Policlinic of Salò, 25087 Brescia, Italy; balzanelli.cristiano@gmail.com; 2 ENT Department—ARNAS Garibaldi of Catania, 95123 Catania, Italy; spataro.daniele.sd@gmail.com; 3 Department of Medical and Surgical Specialties, Radiological Sciences, and Public Health, Section of Audiology, University of Brescia, 25123 Brescia, Italy; 4 Pediatric Otolaryngology Head Neck Surgery Unit, Children Hospital—ASST Spedali Civili of Brescia, 25123 Brescia, Italy

**Keywords:** children, vertigo, benign positional paroxysmal vertigo

## Abstract

The aim of this study was to assess the prevalence and analyze clinical parameters of benign positional paroxysmal vertigo (BPPV) in a pediatric age. A cohort of 423 children under the age of 15 (median age 11. interquartile range 9–13) was submitted to vestibular assessment for balance disorders. Dix-Hallpike and Roll-Supine tests were performed to look for positioning nystagmus using video-infrared goggles. BPPV was found in 43 of 423 children evaluated for balance disorders (10.2%). There were 28 females (65.1%) and 15 (34.9%) males. The posterior canal was involved in 79% of cases and the horizontal canal in 21% of cases. No apogeotropic bilateral or anterior canal form were seen. Thus, BPPV is not an infrequent type of vertigo in children and must be evaluated as soon as possible in order to plan the most appropriate maneuver and restore daily activities as soon as possible, avoiding anxiety and fear.

## 1. Introduction

Benign positional paroxysmal vertigo (BPPV) is a disorder of the vestibular labyrinth consisting of one or more episodes of brief, repeated and severe spinning sensation provoked by changes in posture and head position [1]. It is the most common cause of peripheral vertigo affecting about one-third of adults with balance disorders. The cause of BPPV is related to idiopathic detachment of otoconia from the utricular membrane. The clinical form was well described by Dix and Hallpike in 1952 [2] and since then numerous liberatory maneuvers in both adults and children have been proposed for treatment [3,4,5,6,7,8,9,10,11,12,13]. The prevalence of BPPV in the pediatric age is lower than in adults [12,13,14,15,16,17,18,19,20,21,22,23,24,25,26,27,28,29,30,31,32,33,34,35,36,37,38]. The lower frequency of BPPV in children is due to less common causes of otoconia detachment than in adults (hypertension, metabolic disorders, atherosclerosis) [15]. Providing correct information during the diagnostic and therapeutic assessment is mandatory to avoid fear and panic by children and their parents [39]. Being a disease with possible spontaneous rapid resolution especially in childhood [40,41,42], it is important to obtain an accurate diagnosis as soon as possible, to avoid being left with the diagnostic doubt about a central pathogenesis of symptoms, and to implement effective rehabilitation or pharmacological treatment, avoiding impairment of daily activities.

The aim of our study is to establish the percentage and analyze the clinical features of children with BPPV among a cohort of those suffering from balance disorders observed over a 10-year period.

## 2. Materials and Methods

All children under the age of 15 affected by balance disorders and observed between 2010 and 2019 were included in the study and submitted to our diagnostic work-up. A database was, therefore, developed to collect the parameters on past and present history, bedside, and instrumental explorations of patients.

BPPV bedside screening of all dizzy patients was obtained using unrecorded infrared video-nystagmoscopy (Synapsis Nystalab—wireless video system) to assess eye movements, looking for the following:

- Horizontal and vertical convergence, saccades, smooth pursuit, fixation;
- Spontaneous nystagmus (sitting position);
- Positional nystagmus (supine, left–right side),
- Positioning nystagmus (Dix–Hallpike maneuver, roll-supine test),
- Head shaking test and clinical-head impulse test.

If symptoms persisted and BPPV was excluded, patients could be submitted to other instrumental tests: rotatory test, video head-impulse test (vHIT), vibration-induced nystagmus (VIN), vestibular-evoked myogenic potentials (VEMPs), and posturometric-static platform. We have not described these tests in detail, because our focus is the BPPV condition, but we consider them mandatory for differential purposes when BPPV is not diagnosed. If all the above mentioned clinical and instrumental tests were negative, following the patient’s past/present history and symptoms, in selected cases, we submitted them to other specialistic explorations (i.e., cardiological, ophthalmological, dental, neurological, neuropsychiatric, psychological evaluation, electro-encephalogram, brain CT/MRI). These explorations are not strictly related with BPPV but are essential for differential diagnosis.

BPPV diagnosis was based on nystagmus analysis. Variable latency and fatigability, increasing–decreasing pattern intensity, brief duration (generally within one minute), and geotropism (gravity-changing direction) reversible to the positional change represent the main features of a BPPV nystagmus. Its direction and its consistency with the stimulated canal are crucial in differential diagnosis with other episodic vertigo entities, such as vestibular migraine and benign paroxysmal vertigo of children (BPVC), whose episodes may present with positional vertigo and positional nystagmus mimicking BPPV (even direction changing positional nystagmus) [1]. A geotropic form of posterior canal BPPV was diagnosed when a transient, geotropic, torsional, upbeat nystagmus was observed during the ipsilateral Dix–Hallpike maneuver, performed by bringing the patient from an upright to supine position with the head turned 45° to one side and neck extended 20°. Instead, lateral canal BPPV was diagnosed when the Dix–Hallpike test was negative and horizontal nystagmus is observed bilaterally during the supine roll test and the fast phases of the more intense nystagmus point to the affected side [1,34,43].

Treatment was based on use of the Epley or Semont maneuver for the posterior semicircular canal BPPV [7,44] and the Gufoni maneuver for horizontal semicircular canal BPPV [11].

Data were analyzed using the statistical package SPSS (SPSS Inc., Chicago, IL, USA). The chi-square test was used to compare categorical variables and the Mann–Whitney U test to compare two independent groups on one continuous variable; a *p* < 0.05 was considered statistically significant. Values of continuous variable are expressed as median and interquartile ranges (IQR).

The study was reviewed and approved by the Institutional Ethics Committee.

## 3. Results

A total of 423 participants were included in the study. There were 252 (59.6%) females and 171 (40.4%) males, with a median age at the time of first observation of 11 years (IQR 9–13). In our series, BPPV was the third most frequent cause of vertigo in children, preceded by unilateral vestibular loss (23.9%) and vestibular migraine (21.7%) and followed by benign paroxysmal vertigo in childhood (BPVC) (9.0%), psychogenic vertigo (6.9%), and a miscellaneous of various causes, including dental disorders, ophthalmological disorders, idiopathic vertigo, orthostatic hypotension, otitis media with effusion, epileptic vertigo, postural disorders, post traumatic vertigo, neurological diseases, Ménière’s disease, and bilateral vestibulopathy (Table 1).

Children with BPPV represented 10.2% of all cases of vertigo (43/423), with a median age of 11 years (IQR 8–12): there were 28 females (65.1%) and 15 males (34.9%). The median age was not different from that of the other cumulative causes of vertigo (11 years (IQR 9–13)) (*p* = 0.4).

In addition, the female/male distribution was not different from that of the other cumulative causes of vertigo (58.9% vs. 41.1%) (*p* = 0.4).

In our sample of 43 BPPV patients, there was familial migraine in 10 cases (23.2%), but none manifested migraine symptoms according to International Headache Society Criteria [45]. Nausea and vomiting were present in only 10 cases (23.2%) and 3 manifested previous motion sickness. We had no data regarding objective spontaneous recovery of BPPV, but parents described brief vertigo attacks in children’s past history in 11 cases (25.6%), and we can only surmise that they had previous BPPV episodes, which spontaneously resolved. To optimize the clinical outcome and with prior informed parental consensus, we immediately carried out the liberatory maneuvers at each diagnosis of BPPV. There were 34 cases (79%) of geotropic posterior BPPV. Semont’s maneuver was performed in 33 cases and Epley’s maneuver in 1 case. We had 9 cases (21%) of geotropic horizontal canal BPPV, which were treated by Gufoni’s maneuver. There were no apogeotropic posterior and horizontal canal BPPV, no anterior canal BPPV, and no multi-canalar involvement. Following the first liberatory maneuver, 40 patients (93%) presented complete and stable recovery within 6 months, and 2 patients (4.6%) needed a second treatment for persistence of symptoms (12 days later in one case and 7 days later in the second case). Only one child (2.3%) had recurrence within 3 months who was retreated with sudden stable resolution of BPPV. No patient had post-maneuver residual dizziness, but chronic imbalance before and after repositioning maneuvers was reported by two patients: In one case, the child had BPPV in the ipsilateral side of recurrent vestibular deficit and we hypothesized Lindsay–Hemenway syndrome, without vascular imaging evidence of labyrinthine ischemia; in the other case, the child was anxious and also suffered from episodic panic attacks. BPPV was associated with Pendred Syndrome in one case and with Usher Syndrome in one other case. In our series, only in these two cases did we detect simultaneous auditory involvement with severe bilateral hearing loss. The child affected by Usher syndrome was implanted. We did not observe any relationship of BPPV with neurologic, cardiologic, ophthalmologic, and dental disease. Table 2 shows an outline of neurogenic diseases in our cohort (none affected by BPPV).

## 4. Discussion

The first description of a “positional vertigo” was by Adler in 1897 [46] and in 1952 Dix and Hallpike [2] described a “positional nystagmus of benign type” for the first time. The first communication entitled “Curing BPPV with Liberatory Maneuver” was by Alain Semont in 1983 and published in 1988 [44]. Since then, a rich series of new models and theories on canalar and cupolar lithiasis has been reported, describing many liberatory maneuvers for all semicircular canals, with geotropic and apogeotropic variants, single- and multi-canalar forms, and mono- and bilateral disorders, in both adults and children [3,4,5,6,7,8,9,10,11,12].

BPPV is due to idiopathic canalar or cupolar dislocation of the otoliths, calcium carbonate particles of utricular origin, with abnormal activation of the ampullar receptors. In children, symptoms consist of transitory rotational vertigo and torsional/vertical/horizontal or mixed nystagmus, depending on the position of the intra-labyrinthine otoliths, with latency and intensity in a typical crescendo–decrescendo pattern and it is exhaustible, reversible, fatiguing, similar to adults.

In addition to BPPV, the most frequent forms of vertigo in children are vestibular migraine, BPVC, unilateral vestibular loss, and psychogenic vertigo (Table 1). The extreme variable of published prevalence data on BPPV in children (0.2–21.2% of all balance disorders) (Table 3) reflects the difficulty in carrying out reliable epidemiological studies, in particular due to the inhomogeneous sample (different range of age, diagnostic explorations, statistical methods of data analysis). However, in most studies, BPPV prevalence data are estimated at approximately 5–10% of all causes of pediatric vertigo, for an incidence of about 1% in the pediatric population, compared to 35–37% in adults, showing an increasing percentage proportional to age [13,34]. The posterior semicircular canal is generally involved in about 80% of cases and the lateral semicircular canal in 20% of cases, as in adults. Anterior canal involvement is rare [12,13,14,15,16,17,18,19,20,21,22,32,33,34,35,36,38].

In our sample, 10.2% (43/423) of patients younger than 15 years had a diagnosis of BPPV. These data might be justified by the efficient coordination among different departments in our hospital and thanks to the daily availability of a dedicated Neurotologic Service, which allowed immediate clinical and instrumental evaluation of the dizzy child. This facilitated easier detection of a greater number of pediatric patients with BPPV, who generally tend to quick, complete, and spontaneous recovery in very few days. This observation is generally not reported in the published literature.

In children, the causes of otolithic detachment are less common or absent compared to adults (hypertension, metabolic disorders, and atherosclerosis) [15]. In the medical literature, there is no agreement about the histological aspect of the otoconial membrane. In past studies in the 2000s, some authors attributed the lower incidence of BPPV in children to the lower number of otoliths attached to the cupolas or free in the endolymph [8,14,17], and, in the opinion of other authors, all episodic vertigos in childhood must be considered migraine variants until the age of 11 [16,19]. On the other hand, more recent studies report greater stratification and more otoconial adhesiveness to utricular and saccular macules in childhood than in adults [28].

BPPV is a disease with frequent spontaneous resolution in children, probably due to continuous movements of the head during games and daily physical activity, so that vestibular examination within 24–48 h of the onset of symptoms is mandatory, unless they are frightened by the unusual symptoms [13,42]. Moreover, the children’s innate ability to move and the efficient plasticity of neural pathways allow them to better tolerate vertigo and to rapidly overcome the intensity and duration of symptoms compared with adults [15,42]. This could explain the undescribed post-maneuver residual dizziness in children.

In adults, it is generally accepted spontaneous recovery in an average time of 39 days for the posterior canal and 16 days for the horizontal canal [41], but we have not found similar published data in children, probably due to the difficulty in collecting reliable data at this age [29].

Diagnosis of BPPV in pediatric patients is not simple, as the children can show the typical signs of BPPV during vestibular exploration but report symptoms differently than adults: Their inability to explain symptoms leads caregivers to attempt to interpret symptoms on their behalf, which often leads to inaccurate histories [25]. In fact, small children have a poor vocabulary and are unable to describe their disorder with appropriate terminology and tend to avoid playful activities and isolate themselves from peers [39,42]. Often, they mistake dizziness for fear or report feelings of falling and carousel-like spin [34]. In our series, they sometimes describe a “nightmare” sensation if they awake with vertigo at night. The study of eye movements by Frenzel-oculoscopy or by infrared video-oculoscopy is almost the same as that carried out in adults, as are positional and provocative maneuvers for detection of nystagmus [13,15,26,27,31,38,43]. It is essential to have the child participate during the vestibular evaluation, to attract his attention and avoid excessive concern or lack of cooperation. Parents must be always adequately informed about how and what is being evaluated during the visit, as the unleashing of an intense BPPV with crying and panic of the child during diagnostic or therapeutic maneuvers would lead to excessive alarm.

The differential diagnosis of BPPV in the pediatric age must be adequately aimed towards:

(1) Benign paroxysmal vertigo of childhood, first described by Basser in 1964 [47], characterized by fleeting attacks of vertigo or imbalance lasting seconds or minutes, with quick and complete return to normal activities. It mostly affects children from 3–6 years of age, it is of idiopathic origin, and it is associated with motion sickness as early as 2–3 years of life. It is associated with familial migraine (according to the diagnostic criteria of the International Headache Society) [47], and considered as an early migraine equivalent, such as myogenic torticollis and cyclic vomiting.
(2) Neurological disorders, although rare representing less than 1% of cases, include gliomas, meningiomas, schwannomas, and brainstem–cerebellar neoplasms [13,15,29,38].

## 5. Conclusions

BPPV in a pediatric age is not an infrequent clinical condition (0.2–21.2% in literature, 10.2% in our series).

Our data and published reports confirm the better tolerability of symptoms and possible spontaneous recovery of BPPV in children, due to their neural pathway plasticity and the innate ability to move. Thus, when pediatric BPPV is suspected, it is advisable to perform quick vestibular exploration, plan the most appropriate therapeutic maneuver, and restore the child’s daily activities as soon as possible, avoiding anxiety and fear.

BPPV is a benign condition and the association of children’s BPPV with neurogenic diseases is rare (0% in our series).

## Figures and Tables

**Table 1:** Vertigo in pediatric age: distribution of causes (N = 423).

Disease	Number of Children	Frequency (%)
Unilateral Vestibular Loss	101	23.9
Vestibular migraine	92	21.7
BPPV	43	10.2
BPVC	38	9.0
Psychogenic vertigo	29	6.9
Dental disorders	21	5.0
Ophthalmological disorders	20	4.7
Idiopathic vertigo	15	3.5
Orthostatic hypotension	14	3.3
Otitis media with effusion	13	3.1
Epileptic vertigo	11	2.6
Postural disorders	7	1.7
Post traumatic vertigo	7	1.7
Neurological diseases	5	1.2
Ménière’s disease	4	0.9
Bilateral vestibulopathy	3	0.7

BPPV: benign paroxysmal positional vertigo. BPVC: benign paroxysmal vertigo of children.

**Table 2:** Neurogenic diseases associated with vertigo in our cohort.

Sex	Age	Diagnosis	Subjective Symptoms	Bedside Signs	Instrumental Tests	Other Exploration
M	14	Neuromotor development delay and previous stroke	Chronic Dizziness	Ataxia, inccordination, no nystagmus	Not collaborative	MRI, neuropsychiatric, neurologic and speech therapist evaluation
M	12	Outcome of brain trauma	Serious imbalance and episodic falls	Persistent right irregular horizontal nystagmus	Not collaborative	MRI, neurologic revaluation
M	9	NF1, Arnold Chiari type1, idrocefalo, left horizontal canal deficit	Imbalance	Right HST	Left horizontal canal deficit	MRI, neurologic evaluation
F	5	Levo-Dopa responsive dystonia	Imbalance	No nystagmus	Normal	MRI, neurologic, cardiologic, blood, physiatrist evaluations
M	14	Cornelia De Lange	Brief imbalance episodes and falls	No nystagmus	Normal	MRI

**Table 3:** Prevalence of BPPV in children.

Reference	Year	Sample (n°)	BPPV Prevalence (%)
Russell and Abu-Arafeh [16]	1999	2165	2.6
Choung et al. [18]	2003	55	3.6
Riina et al. [20]	2005	110	1
Erbek et al. [21]	2006	50	12
Balatsouras et al. [22]	2007	54	7.3
Szirmai [24]	2010	145	21.2
Jahn et al. [26]	2011	not specified (review)	5
Saka et al. [29]	2013	3341	3
Sommerfleck et al. [31]	2016	216	3.9
Lee et al. [32]	2017	411 (multi-center)	5.1
Messina et al. [33]	2017	2682 (multi-center)	0.2
Brodsky et al. [34]	2017	110	19.8
Wiener-Vacher et al. [35]	2018	2528	1.2
Choi et al. [37]	2020	20	9.5
Davitt et al. [38]	2020	2726 (review)	2.6
Present study	2021	423	10.2

## Data Availability

The data presented in this study are available on request from the corresponding author. The data are not publicly available due to underage subjects.

## References

[1] Von Brevern M., Bertholon P., Brandt T., Fife T., Imai T., Nuti D., Newman-Toker D. (2015). Benign paroxysmal positional vertigo: Diagnostic criteria. J. Vestib. Res..

[2] Dix R., Hallpike C.S. (1952). The pathology, symptomatology and diagnosis of certain common disorders of the vestibular system. Ann. Otol. Rhinol. Laryngol..

[3] McClure J. (1985). Horizontal canal BPV. J. Otolaryngol..

[4] Cipparrone L., Corridi G., Pagnini P., Dufour A. (1985). Cupulolitiasi. Atti della V Giornata Italiana di Nistagmografia Clinica, San Marino, 13 Aprile 1985.

[5] Katsarkas A. (1987). A nystagmus of paroxysmal positional vertigo: Some new insights. Ann. Otol. Rhinol. Laryngol..

[6] Pagnini P., Nuti D., Vannucchi P. (1989). Benign paroxysmal vertigo of the horizontal canal. ORL J. Otorhinolaryngol. Relat. Spec..

[7] Epley J.M. (1992). The canalith repositioning procedure: For treatment of benign paroxysmal positional vertigo. Otolaryngol. Head Neck Surg..

[8] Herdman S.J., Tusa R.J., Zee D.S., Proctor L.R., Mattox D.E. (1993). Single treatment approaches to benign paroxysmal positional vertigo. Arch. Otolaryngol. Head Neck Surg..

[9] Baloh R.W. (1994). Horizontal benign positional vertigo. Neurology.

[10] Vannucchi P., Giannoni B., Pagnini P. (1997). Treatment of horizontal semicircular canal benign paroxysmal positional vertigo. J. Vestib. Res..

[11] Gufoni M., Mastrosimone L., Di Nasso F. (1998). Repositioning maneuver in benign paroxysmal vertigo of horizontal semicircular canal. Acta Otorhinolaryngol. Ital..

[12] Marcelli V., Piazza F., Pisani F., Marciano E. (2006). Neuro-otological features of benign paroxysmal vertigo and benign paroxysmal positioning vertigo in children: A follow-up study. Brain Dev..

[13] Libonati G.A. (2017). La vertigine in età pediatrica. Prospett. Pediatr..

[14] Igarashi M., Saito R., Mizukosh K., Alford B.R. (1993). Otoconia in young and elderly persons: A temporal bone study. Acta Otolaryngol. (Stockh).

[15] D’Agostino R., Tarantino V., Melagrana A., Taborelli G. (1997). Otoneurologic evaluation of child vertigo. Int. J. Pediatr. Otorhinolaryngol..

[16] Russell G., Abu-Arafeh I. (1999). Paroxysmal vertigo in children: An epidemiological study. Int. J. Pediatr. Otorhinolaryngol..

[17] Bachor E., Wright C.G., Karmody C.S. (2002). The incidence and distribution of cupular deposits in the pediatric vestibular labyrinth. Laryngoscope.

[18] Choung Y.H., Park K., Moon S.K., Kim C.H., Ryu S.J. (2003). Various causes and clinical characteristics in vertigo in children with normal eardrums. Int. J. Pediatr. Otorhinolaryngol..

[19] Uneri A. (2003). Evaluation of vestibular functions in children with vertigo attacks. Arch. Dis. Child..

[20] Riina N., Ilmari P., Kentala E. (2005). Vertigo and imbalance in children: A retrospective study in a Helsinki University Otorhinolaryngology Clinic. Arch. Otolaryngol. Head Neck Surg..

[21] Erbek S.H., Erbek S.S., Yilmaz I., Topal O., Ozgirgin N., Ozluoglu L.N., Alehan F. (2006). Vertigo in childhood: A clinical experience. Int. J. Pediatr. Otorhinolaryngol..

[22] Balatsouras D.G., Kaberos A., Assimakopoulos D., Katotomichelakis M., Economou N.C., Korres S.G. (2007). Etiology of vertigo in children. Int. J. Pediatr. Otorhinolaryngol..

[23] Neuhauser H.K. (2007). Epidemiology of vertigo. Curr. Opin. Neurol..

[24] Szirmai A. (2010). Vestibular disorders in childhood and adolescents. Eur. Arch. Otorhinolaryngol..

[25] Cho E.I., White J.A. (2011). Positional vertigo: As occurs across all age groups. Otolaryngol. Clin. N. Am..

[26] Jahn K., Langhagen T., Schroeder A.S., Heinen F. (2011). Vertigo and dizziness in childhood—Update on diagnosis and treatment. Neuropediatrics.

[27] Dispenza F., De Stefano A. (2012). Vertigo in childhood: A methodological approach. Bratisl. Lek. Listy.

[28] Shetye A. (2012). Benign paroxysmal positional vertigo in a child: An infrequent complication following a fairground ride and post-cochlear implant surgery. Cochlear Implants Int..

[29] Saka N., Imai T., Seo T., Ohta S., Fujimori K., Masumura C., Inohara H., Sakagami M. (2013). Analysis of benign paroxysmal positional nystagmus in children. Int. J. Pediatr. Otorhinolaryngol..

[30] Li C.M., Hoffman H.J., Ward B.K., Cohen H.S., Rine R.M. (2016). Epidemiology of dizziness and balance problems in children in the United States: A population based study. J. Pediatr..

[31] Sommerfleck P.A., Gonzalez Macchi M.E., Weinschelbaum R., De Bagge M.D., Bernaldez P., Carmona S. (2016). Balance disorders in childhood: Main etiologies according to age. Usefulness of the video head impulse test. Int. J. Pediatr. Otorhinolaryngol..

[32] Lee J.D., Kim C.H., Hong S.M., Kim S.H., Suh M.W., Kim M.B., Shim D.B., Chu H., Lee N.H., Kim M. (2017). Prevalence of vestibular and balance disorders in children and adolescents according to age: A multi-center study. Int. J. Pediatr. Otorhinolaryngol..

[33] Messina A., Casani A.P., Manfrin M., Guidetti G. (2017). Italian survey on benign paroxysmal positional vertigo. Acta Otorhinolaryngol. Ital..

[34] Brodsky J.R., Lipson S., Wilber J., Zhou G. (2018). Benign Paroxysmal Positional Vertigo (BPPV) in children and adolescents: Clinical Features and response to therapy in 110 pediatric patients. Otol. Neurotol..

[35] Wiener-Vacher S.R., Quarez J., Le Priol A. (2018). Epidemiology of vestibular impairments in a pediatric population. Semin. Hear..

[36] Hain T., Cherchi M. (2019). Migraine associated vertigo. Adv. Otorhinolaryngol..

[37] Choi H.G., Kim G., Kim B.J., Hong S.K., Kim H.J., Lee H.J. (2020). How rare is benign paroxysmal positional vertigo in children? A review of 20 cases and their epidemiology. Int. J. Pediatr. Otorhinolaryngol..

[38] Davitt M., Delvecchio M.T., Stephen C. (2020). The differential diagnosis of vertigo in children: A systematic review of 2726 cases. Pediat. Emerg. Care.

[39] Medeiros I.R., Bittar R.S., Pedalini M.E., Lorenzi M.C., Formigoni L.G., Bento R.F. (2005). Vestibular rehabilitation therapy in children. Otol. Neurotol..

[40] D’Agostino R., Melagrana A., Taborelli G. (2003). Benign positional paroxysmal vertigo of horizontal semicircular canal in the child: Case report. Int. J. Pediatr. Otorhinolaryngol..

[41] Imai T., Ito M., Takeda N., Uno A., Matsunaga T., Sekine K., Kubo T. (2005). Natural course of the remission of vertigo in patients with benign paroxysmal positional. Neurology.

[42] Casani A.P., Dallan I., Navari E., Sellari Franceschini S., Cerchiai N. (2015). Vertigo in childhood: Proposal for a diagnostic algorithm based upon clinical experience. Acta Otorhinolaryngol. Ital..

[43] Bhattacharyya N., Gubbels S.P., Schwartz S.R., Edlow J.A., El-Kashlan H., Fife T., Holmberg J.M., Mahoney K., Hollingsworth D.B., Roberts R. (2017). Clinical practice guideline: Benign paroxysmal positional vertigo (Update). Otolaryngol. Head Neck Surg..

[44] Semont A., Freyss G., Vitte E. (1988). Curing the BPPV with a liberatory manoeuvre. Adv. Otorhinolaryngol..

[45] Headache Classification Committee of the International Headache Society (IHS) (2018). The international classification of headache disorders, 3rd edition. Cephalalgia.

[46] Adler A. (1897). Über den “einseitigen Drehschwindel”. Dtsch. Z. Nervenheilkd..

[47] Basser L.S. (1964). Benign paroxysmal vertigo of childhood: A variety of vestibular neuronitis. Brain.

